# The synaptic receptor Lrp4 promotes peripheral nerve regeneration

**DOI:** 10.1038/s41467-018-04806-4

**Published:** 2018-06-19

**Authors:** Katherine D. Gribble, Lauren J. Walker, Louis Saint-Amant, John Y. Kuwada, Michael Granato

**Affiliations:** 1 Department of Cell and Developmental Biology, Perelman School of Medicine, University of Pennsylvania, Philadelphia, PA 19104 USA; 2 Department of Molecular, Cellular, and Developmental Biology, University of Michigan, Ann Arbor, MI 48109 USA

## Abstract

Early during PNS regeneration, regenerating axons emerge from the proximal nerve stump, yet whether they extend simultaneously or whether pioneering axons establish a path for follower axons remains unknown. Moreover, the molecular mechanisms underlying robust regeneration are incompletely understood. Using live imaging, we demonstrate that in zebrafish pioneering axons establish a regenerative path for follower axons. We find this process requires the synaptic receptor *lrp4*, and in *lrp4* mutants pioneers are unaffected while follower axons frequently stall at the injury gap, providing evidence for molecular diversity between pioneering and follower axons in regeneration. We demonstrate that Lrp4 promotes regeneration through an axon extrinsic mechanism and independent of membrane anchoring and MuSK co-receptor signaling essential for synaptic development. Finally, we show that Lrp4 coordinates the realignment of denervated Schwann cells with regenerating axons, consistent with a model by which Lrp4 is repurposed to promote sustained peripheral nerve regeneration via axon-glia interactions.

## Abstract (web summary)

Axons of the peripheral nervous system regenerate along Schwann cells following injury. Here, the authors show that regenerating axons follow pioneer axons and this process is mediated by lipoprotein receptor-related protein 4 (Lrp4), a protein otherwise known for its role in synapse formation.

## Introduction

Axons of the peripheral nervous system can regenerate after injury^1^, yet the molecular mechanisms that promote robust nerve regeneration are not fully understood. After sustaining an injury, peripheral nerves initiate the program of Wallerian degeneration that causes self-destruction of distal axons^2^. Distal axon debris is subsequently removed by macrophages and Schwann cells^3–6^, clearing the path along which axons can regrow. Axonal regeneration begins when growth cones sprout from the proximal nerve stump and stabilize into growing axons, and the current view is that denervated Schwann cells in the distal nerve stump become activated and provide diffusible factors, including NGF, BDNF, GDNF, and FGF that promote growth cone sprouting, as well as axonal growth and guidance^7–10^. Although there is evidence that axonal regrowth is staggered^11,12^, it is unclear whether axons emerge in waves from the nerve stump and fan out in search of their original trajectory, or whether a limited number of axons pioneer a path that later emerging follower axons then fasciculate with to traverse the injury site and grow back toward their original targets.

Shortly after sprouting from the proximal nerve stump, regenerating axons grow toward and along denervated/activated Schwann cells^3,13–15^. Schwann cells realign with regenerating axons, and their morphology changes dramatically as they revert from an activated, regeneration-supporting Schwann cell to a pre-injury myelinating Schwann cell^1,3,16^. Several molecular pathways critical for Schwann cells to transition from a myelinating Schwann cell to an activated, denervated and more immature Schwann cell have been documented^17–20^. In contrast, the mechanisms underlying the realignment of denervated Schwann cells with regenerating axons, and the mechanisms that trigger this pronounced change in Schwann cell morphology as they revert to a pre-injury myelinating Schwann cell are not well understood.

Here we use live cell imaging in larval zebrafish and demonstrate that upon complete peripheral nerve transection, individual axons emerging from the proximal stump pioneer a path across the injury gap. Later-emerging axons fasciculate with these pioneer axons to cross the injury gap and to return toward their original synaptic targets. We find that this process requires the synaptic low-density lipoprotein receptor-related protein 4 (Lrp4), and that in *lrp4* null mutants^21^ pioneer axons are unaffected while follower axons frequently fail to cross the injury gap and stall. Moreover, we show that *lrp4* promotes regeneration through an axon extrinsic mechanism, and independently from its membrane anchor and without signaling through the canonical Agrin/MuSK/Rapsyn signaling pathway. Instead, *lrp4* coordinates the realignment of regenerating axons with denervated Schwann cells. Together, our findings demonstrate the existence of and the molecular diversity between axonal pioneers and followers in regeneration, and reveal an unexpected in vivo function for *lrp4* in peripheral nerve regeneration.

## Results

### Regenerating pioneer axons mark a path for follower axons

During development, subsets of neurons extend pioneering axons that outline the trajectory for later emerging follower axons^22–26^. Whether regenerating axons employ a similar strategy of pioneer and follower axons or whether they emerge from the proximal nerve stump in waves and fan out searching for their original trajectory is currently unknown. We have previously shown that laser-mediated transection of spinal motor nerves in 5 day post fertilization (dpf) zebrafish larvae results in robust axonal regeneration within 48 h post transection^27–29^. Importantly, we find that regeneration in larval zebrafish is characterized by key features of vertebrate peripheral nerve regeneration, including the ability of *Wld*^*s*^ to delay axonal fragmentation and the dependence on Schwann cells for successful regeneration^27,28^.

**Fig. 1:**
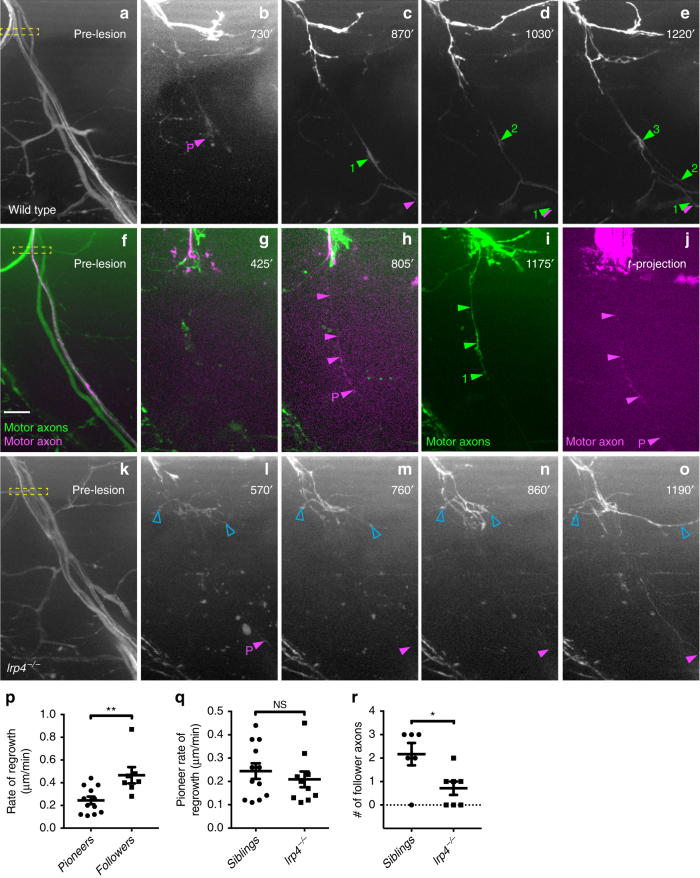
Pioneering axons establish regenerative path for follower axons. **a**–**e** Images from time lapse movie showing early regeneration of wild-type motor nerve with all axons expressing GFP. **a** Pre-lesion image; yellow box marks transection site. **b** A pioneering axon (magenta arrowhead and ‘P’) crosses the injury gap ~800 min post-injury and extends ventrally. **c**, **d**, **e** Follower axons fasciculate with pioneering axon (numbered green arrowheads). **f**–**j** Still images from time lapse movie showing early regeneration of 5 dpf wild-type motor nerve; all axons express GFP, single-axon expresses mKate (magenta). **f** Pre-lesion image; yellow box marks transection site. Scale bar is 10 µm. **g** Axon sprouts in proximal nerve stump start extending and retracting ~400 min post transection. **h** Single-pioneering axon (magenta) crosses the injury gap and extends ventrally along distal nerve by 800 min post transection. Magenta arrowheads mark pioneer axon, which grows at a rate of 0.27 µm/min. **i** Follower axon extends and grows along pioneering axon; green arrowheads mark follower path, which grows at a rate of 0.36 µm/min. **j** By the end of imaging, the magenta pioneer axon has extended ventrally, as shown via a maximum projection image across time (magenta arrowheads). **k**–**o** Still images from time lapse movie showing early regeneration of *lrp4* mutant motor nerve labeled with GFP. **k** Pre-lesion image; yellow box marks transection site. **l** Early growth cones sprout from proximal stump (open blue arrowheads) and pioneer axon extends ventrally ~600 min post transection (magenta arrowhead and ‘P’). **m**–**o** Throughout imaging, only the pioneer axon extends to ventral myotome; follower axons explore transection site but do not traverse injury gap (open blue arrowheads). **p** Quantification of pioneer axon growth rate and follower axon growth rate in wild-type siblings. Pioneer axon average rate of regrowth is 0.24 µm/min (*n* = 12 nerves in 8 larvae); follower axon average rate of regrowth is 0.47 µm/min (*n* = 7 nerves in 4 larvae; unpaired *t*-test *p* = 0.0055; *t* = 3.181, df = 17; error bars show mean and SEM). **q** Quantification of pioneer axon rate of regrowth in siblings and *lrp4* mutants. Pioneer axons grow at equivalent rates: sibling average rate of regrowth = 0.24 µm/min (*n* = 12 nerves in 8 larvae); *lrp4*^*−/−*^ average rate of regrowth = 0.21 µm/min (*n* = 10 nerves in 4 larvae; unpaired two-tailed *t*-test *p* = 0.4702, *t* = 0.7361, df = 20; error bars show mean and SEM). **r** Number of follower axons in siblings and *lrp4* mutants. In siblings, multiple follower axons extend (average number of follower axons = 2.2, *n* = 6 nerves in 2 larvae) while in *lrp4* mutants, follower axons fail to extend ventrally (*lrp4* mutant average number of follower axons = 0.7, *n* = 7 nerves in 2 larvae). Number of follower axons in siblings versus mutants is significantly different (two-tailed *t*-test *p* = 0.0206, *t* = 2.703, df = 11; error bars show mean and SEM)

Spinal motor nerves consist of ~60 individual axons, and to examine whether regenerating axons emerge simultaneously or whether they emerge in a temporal sequence, we transected individual spinal nerves in the *mnx1:GFP* transgenic line in which all spinal motor axons express GFP. Time lapse imaging revealed that starting around 9 h post transection (hpt) between two and six axon sprouts emerge from the proximal stump, rapidly extending and retracting (Supplementary Movie 1 and ref.^28^). Around 11 hpt, what appeared to be a single axon stabilized and began to pioneer a path across the transection gap (Fig. 1b; magenta arrowhead and ‘P’). At around 18 hpt, the first ‘follower’ axon (Fig. 1c; green arrowhead and ‘1’) emerged from the proximal stump and grew alongside the pioneer axon. Within the next 3–4 h up to three additional axons emerged and grew along the earlier axons across the injury gap and toward their original targets (average 2.2 followers; Fig. 1d, e, r and Supplementary Movie 1). We noticed that the early, pioneering axons extend at an average rate of 0.24 µm per minute, while later emerging follower axons extend almost twice as fast (0.47 µm per minute; Fig. 1p).

To confirm that indeed a single axon pioneered the regenerative path, we used a sparse labeling strategy^30^ to genetically label individual neurons and their axons with mKate (magenta, Fig. 1f) in the background of the *mnx1:GFP* transgenic line labeling all motor axons. Prior to nerve transection, we selected spinal segments in which only one motor neuron and its axon were labeled with mKate, thereby ensuring single-axon resolution (magenta, Fig. 1f). In the majority of cases, we observed an individual mKate-positive axon extending along GFP-positive axons that had emerged from the proximal nerve stump earlier. In a small number of instances individual mKate-positive axons were the first to emerge from the proximal nerve stump and pioneered a regenerative path, followed by GFP-only expressing axons (Fig. 1h–j, Supplementary Movie 2). Thus, regenerating axons segregate into a larger pool of fast growing follower axons that tightly fasciculate with a single axon that successfully pioneered a path across the injury site.

### *lrp4* promotes nerve regeneration axon-extrinsically

We next sought to gain insights into the molecular mechanisms that promote peripheral nerve regeneration. For this we examined a collection of existing mutant lines harboring mutations in genes critical for axonal growth and guidance, as well as in synapse formation. This revealed an unexpected role for the synaptic low-density lipoprotein receptor-related protein 4 (Lrp4). Lrp4 encodes a single-pass transmembrane domain protein best known for its evolutionarily conserved role in neuromuscular synapse development^21,31^. Live cell imaging following nerve transection revealed that like in wild-type animals (Fig. 1a–j and Supplementary Movies 1 and 2), pioneer axons in *lrp4* mutants emerged from the proximal nerve stump starting around 10 hpt, and navigated across the transection gap and into the ventral myotome with growth rates indistinguishable from those in wild-type siblings (Fig. 1k–o, q and Supplementary Movie 3). In contrast to wild-type animals, follower axons in *lrp4* mutants remained in a state of exploratory extension and retraction, ultimately stalling at the injury gap (Fig. 1k–o, r; open blue arrowheads; mean number of followers observed during imaging window = 0.7 axons; *n* = 7 nerves). Consequently, this reduced the number of follower axons that crossed the injury gap within the first 20 hpt by more than threefold, from an average of 2.2–0.7 follower axons per nerve (Fig. 1r). Importantly, motor axonal outgrowth during development is unaffected in *lrp4* mutants^21^, demonstrating a specific role for Lrp4 during axonal regeneration. Thus, while *lrp4* appears dispensable for regenerative growth of pioneer axons, it is critical for follower axons to robustly regrow across the injury gap and towards their original targets.

**Fig. 2:**
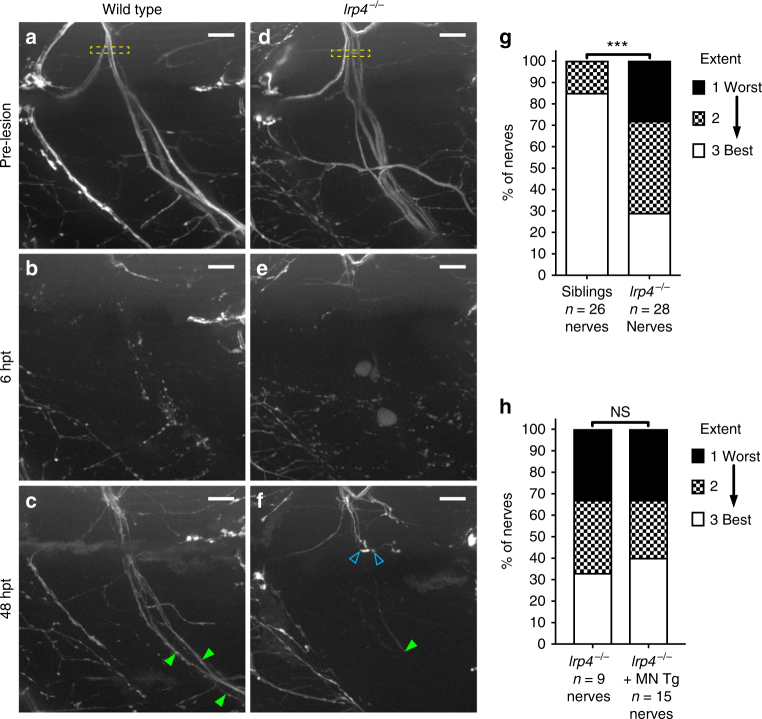
*lrp4* is required non-cell-autonomously for axonal growth during regeneration. **a** Lateral view of a wild-type motor nerve labeled with GFP before laser transection at 5 dpf, with transection site marked by yellow box. Scale bar is 10 µm. **b** Six hours post transection (hpt), motor axons distal to the transection site have fully degenerated. **c** Forty-eight hours post transection, motor axons have regrown along their original path; green arrowheads indicate multiple regenerated fascicles; this representative nerve received a score of ‘best’ regeneration. **d** Lateral view of *lrp4* mutant motor nerve labeled with GFP before laser transection at 5 dpf, with transection site marked by yellow box. **e** Six hours post transection, distal motor axons have fully degenerated. **f** Forty-eight hours post transection, motor axons have sprouted from the proximal stump but most fail to cross the injury site (open blue arrowheads); green arrowhead indicates stalled fascicle; this representative nerve received a score of ‘worst’ regeneration. **g** Quantification of nerve regeneration in wild type and *lrp4* mutant larvae. In wild type, 85% of nerves regenerate “best” with two or more distinct fascicles (*n* = 26 nerves from 13 larvae). In *lrp4* mutants, 70% of nerves regenerate zero or one fascicle (*n* = 28 nerves from 13 larvae) by 48 h post transection. Wild-type siblings regenerate significantly better than *lrp4* mutants (*χ*^2^-test *p* < 0.0001, *χ*^2^ = 18.48, df = 2). **h** Quantification of nerve regeneration in *lrp4* mutant larvae and *lrp4* mutant larvae expressing the motor neuron-specific *mnx1:Lrp4-GFP* transgene. At 48 hpt, *lrp4* mutant larvae expressing *Tg(mnx1:Lrp4-GFP)* regenerate poorly (*n* = 15 nerves from 6 larvae), like *lrp4* mutant larvae (*n* = 9 nerves from 4 larvae; *χ*^2^-test *p* = 0.9266, *χ*^2^ = 0.1524, df = 2)

We next wondered whether the reduced ability of follower axons to cross the injury gap was transient or diminished the overall robustness of peripheral nerve regeneration. Prior to nerve transection at 5 days post fertilization (dpf), individual motor nerves in wild type and *lrp4* mutants are comprised of about 60 axons organized into 3–4 major fascicles that extend into the ventral myotome (Fig. 2a, d)^27,28^. By 48 h post transection, wild-type axons have robustly regenerated and form several fascicles that extend back towards their original targets in the ventral myotome (Fig 2c; green arrowheads; quantified in 2g). In contrast, *lrp4* mutants display a marked reduction in the number of axon fascicles that have reached the ventral myotome, concomitantly with a marked increase of fascicles stalling prematurely (Fig 2f; open blue arrowheads; quantified in 2g).

Finally, we asked whether the inability of the follower axons to grow alongside the pioneer axon in *lrp4* mutants is the consequence of a motor neuron intrinsic defect, for example an axonal fasciculation defect. To test this possibility we first generated transgenic lines that express a Lrp4-GFP fusion protein under a muscle-specific promoter or under a motor neuron-specific promoter. Transgenic expression of *lrp4* in skeletal muscle restored the formation of neuromuscular junctions in otherwise *lrp4* mutant animals, however did not rescue the regeneration phenotype (Supplementary Fig. 1). In contrast, transgenic expression of Lrp4-GFP in spinal motor neurons failed to rescue the motor axon regeneration defect observed in these mutants (Fig 2h), consistent with the notion that *lrp4* acts through a cell non-autonomous, extrinsic mechanism. Thus, early during the regeneration process *lrp4* acts through an extrinsic mechanism to selectively promote the growth of follower axons alongside the pioneering axons, thereby promoting robust peripheral nerve regeneration in vivo.

### *lrp4* realigns regenerating axons with Schwann cells

**Fig. 3:**
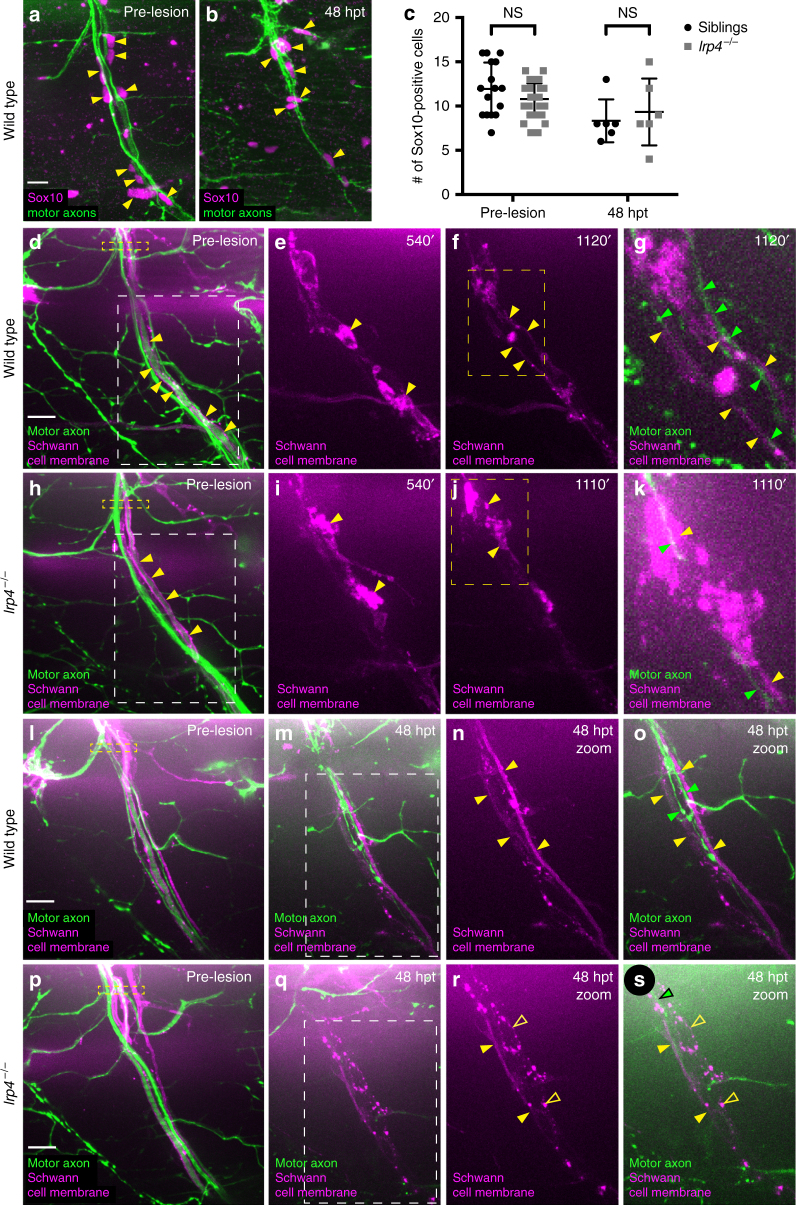
Schwann cell morphology during early regeneration is disrupted in *lrp4* mutants. **a** Pre-transection 5 dpf, GFP-labeled sibling nerve stained with anti-Sox10 antibody labeling Schwann cell nuclei (magenta). Yellow arrowheads mark Schwann cell nuclei; scale bar 10 µm. **b** Sibling nerve 48 hpt labeled with GFP and stained with anti-Sox10 antibody. Yellow arrowheads mark Schwann cell nuclei. **c** Quantification of Schwann cell nuclei along individual nerves pre-transection and 48 hpt in siblings and *lrp4* mutants. Pre-transection average Schwann cell number in siblings = 11.9 per nerve, *n* = 16 nerves; average Schwann cell number in *lrp4* mutants = 10.78 per nerve, *n* = 36 nerves (two-tailed *t*-test *p* = 0.08, *t* = 1.743, df = 50; error bars denote mean and SD). Average Schwann cell number 48 hpt in siblings = 8.3 per nerve, *n* = 6 nerves; average Schwann cell number 48 hpt in *lrp4* mutants = 9.3 per nerve, *n* = 6 nerves (two-tailed *t*-test *p* = 0.59, *t* = 0.5459, df = 10; error bars denote mean and SD). **d**–**k** Images from time lapse movies showing early regeneration dynamics in sibling (**d**–**g**) and *lrp4* mutant (**h**–**k**) nerves. Motor axons express GFP, Schwann cells express mRFP (magenta). **d** Pre-transection sibling nerve, yellow box marks transection site. Schwann cell membranes (yellow arrowheads) are thin and closely associate with motor axons. White box indicates region magnified in (**e**–**f**). Scale bar 10 µm. **e** Magnified image showing granular Schwann cell morphology characteristic of a denervated Schwann cell (yellow arrowheads). **f** Schwann cell membranes revert to smooth pre-injury morphology (yellow arrowheads). Yellow box marks region magnified in **(g**). **g** Magnified image at same timepoint as (**f**), showing regenerating axons (green arrowheads) associating with flattened Schwann cell membranes (yellow arrowheads; *n* = 4/4 nerves). **h** Pre-transection *lrp4* mutant nerve, yellow box marks transection site. Schwann cell membranes (yellow arrowheads) look wild type. White box indicates region magnified in (**i**–**j**). **i** Magnified image showing granular Schwann cell morphology (yellow arrowheads). **j** *Lrp4* mutant Schwann cell membranes fail to revert to smooth pre-injury morphology (yellow arrowheads). Yellow box marks region magnified in (**k**). **k** Magnified image at same timepoint as **j**, showing a regenerating axon (green arrowhead) associating with granular Schwann cell membranes (yellow arrowheads; *n* = 7/8 nerves). **l** Pre-transection sibling nerve; yellow box marks transection site. **m**–**o** By 48 hpt, Schwann cell membranes form two thin, continuous tracks along which regenerating axons grow; white box indicates region magnified in **n**–**o** (filled yellow arrowheads mark Schwann cell membranes, green arrowheads mark regenerating axons; *n* = 23/24 nerves). **p** Pre-transection *lrp4* mutant nerve; yellow box marks transection site. **q**–**s** By 48 hpt, Schwann cell membranes fail to form two thin, continuous tracks and remain granular; white box in (**q**) indicates the region magnified in **r**–**s** (filled yellow arrowheads mark continuous Schwann cell membrane track; open yellow arrowheads mark granular membranes; green arrowhead marks regenerating axon; *n* = 18/24 nerves)

Schwann cells and perineural glia cells have both been shown to play critical roles in peripheral nerve regeneration^32–34^. For example, in rodents EphB signaling promotes Schwann cell migration and axonal regrowth across the injury site^35^. Similarly, in zebrafish lacking Schwann cells, regenerating axons sprout from the proximal nerve stump but fail to grow across the injury gap^28^, reminiscent of the phenotype we observe in *lrp4* mutants. Given the prominent role of Schwann cells in peripheral nerve regeneration, we examined Schwann cell numbers and morphology before and after injury. We found that in *lrp4* mutants the number of Sox10-positive Schwann cells associated with individual motor nerves before transection and 48 h post transection was equivalent to those in wild-type animals (Fig. 3a–c), strongly arguing that Lrp4 is dispensable for Schwann cell development and survival.

We then examined whether *lrp4* is important for the stereotyped morphological changes associated with injury-induced Schwann cell activation. For this we utilized a combination of stable transgenic lines in which both motor axons and Schwann cell membranes are labeled^36^. We have previously shown that before injury Schwann cell membranes ensheath individual motor axons^27^, and that following nerve transection when axons start to fragment, Schwann cell membranes reorganize, changing from a smooth, tube-like appearance to a more rounded and granular morphology, indicative of their transition to an activated, dedifferentiated state (Fig. 3e)^28^. Prior to injury, Schwann cell morphology in *lrp4* mutants was indistinguishable from that in wild-type siblings (Fig. 3d, h). Live cell imaging between 10 and 24 hpt revealed that in *lrp4* mutants Schwann cell morphology changed upon injury indistinguishable from the process in wild-type siblings (Fig. 3e, i; yellow arrowheads). In wild-type larvae Schwann cell membranes re-extend and become smoother to resemble their pre-transection morphology around 19 hpt, just as regenerating axons are contacting the Schwann cells (Fig. 3f, g, Supplementary Movie 4; Schwann cell membranes marked by yellow arrowheads, axons marked by green arrowheads; *n* = 4 out of 4 nerves). By 48 hpt, when several axon fascicles have regenerated to their muscle targets, Schwann cell membranes were even straighter and more continuous than at 24 hpt, typically forming two discrete tracks along which growing fascicles navigate (Fig. 3l–o; continuous Schwann cell membranes marked by filled yellow arrowheads; *n* = 23 out of 24 nerves). In contrast, in *lrp4* mutants Schwann cells appear unable to transition back to their pre-injury morphology and instead retain a rounded and granular morphology, even despite apparent contacts with a pioneering axon (Fig. 3j, k, Supplementary Movie 5; Schwann cell membranes marked by yellow arrowheads, axons marked by green arrowheads; *n* = 7 out of 8 nerves). At 48 hpt, *lrp4* mutant Schwann cells retained their more granular morphology, and the majority failed to form two distinct tracks (Fig. 3p–s; continuous Schwann cell membrane marked by filled yellow arrowheads, granular membranes marked by open yellow arrowheads, motor axon marked with green arrowhead; *n* = 18 out of 24 nerves). Thus, while dispensable for Schwann cell development, *lrp4* promotes the morphological changes associated with Schwann cell re-differentiation after injury-induced de-differentiation.

### Lrp4 promotes regeneration independently of MuSK signaling

**Fig. 4:**
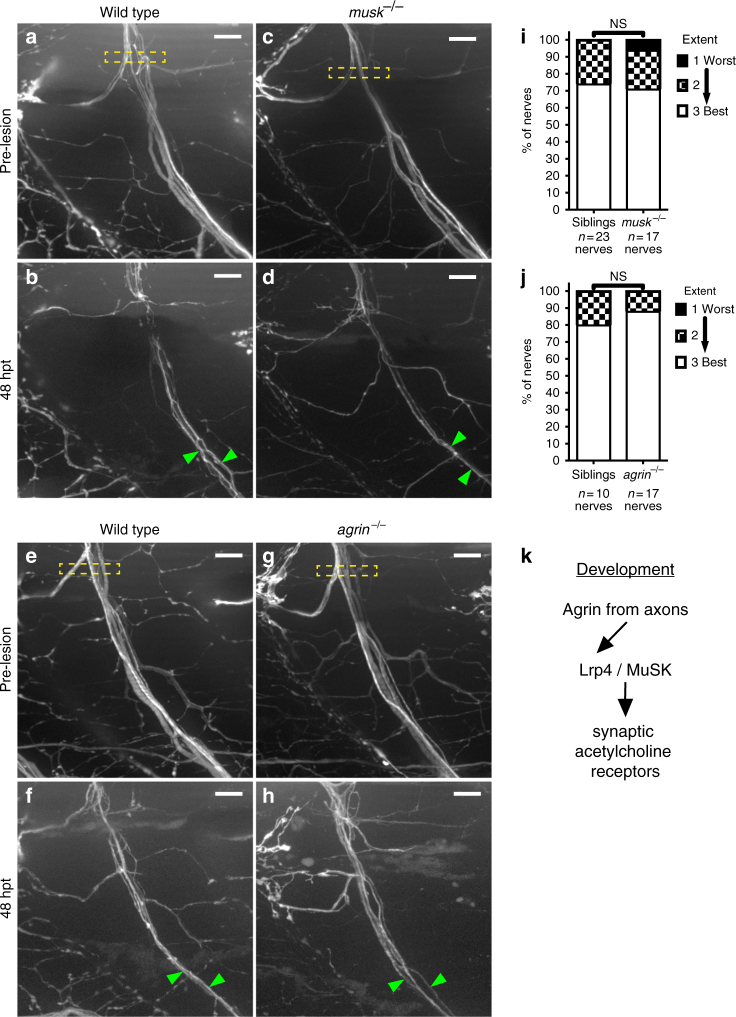
*lrp4* acts in a MuSK-independent pathway to promote axonal regeneration. **a** Lateral view of wild-type GFP-labeled motor nerve before laser transection at 5 dpf. Yellow dashed box indicates transection site, scale bar is 10 µm. **b** Forty-eight hours post transection, multiple axon fascicles have regenerated ventrally. Green arrowheads indicate regenerated fascicles. **c** Lateral view of *musk* mutant motor nerve before laser transection at 5 dpf. Yellow dashed box indicates transection site. **d** Forty-eight hours post transection, motor axons have regenerated ventrally. Green arrowheads indicate regenerated fascicles. **e** Lateral view of wild-type GFP-labeled motor nerve before transection. **f** Forty-eight hours post transection, multiple axon fascicles regenerate ventrally. Green arrowheads mark regenerated fascicles. **g** Lateral view of *agrin* mutant motor nerve before transection at 5 dpf. Yellow box indicates transection site. **h** Forty-eight hours post transection, *agrin* mutant motor axons have regenerated ventrally. Green arrowheads mark regenerated fascicles. **i** Quantification of nerve regeneration in wild-type siblings (*n* = 23 nerves from 11 larvae) and *musk* mutants (*n* = 17 nerves from 11 larvae) at 48 h post transection. *musk* mutant motor nerves regenerate as well as wild-type sibling motor nerves (Fisher’s exact test *p* = 0.4982). **j** Quantification of nerve regeneration in wild-type siblings (*n* = 10 nerves from 4 larvae) and *agrin* mutants (*n* = 17 nerves from 6 larvae). *Agrin* mutant motor nerves regenerate as well as wild-type sibling nerves (Fisher’s exact test *p* = 0.6125). **k** Schematic of the canonical Agrin-Lrp4-MuSK pathway critical for neuromuscular synapse formation

We next wanted to decipher the molecular mechanisms by which Lrp4 promotes peripheral nerve regeneration, and one obvious signaling pathway is the Agrin/MuSK pathway required for vertebrate neuromuscular synapse development^37^. There, Lrp4 expressed on the surface of skeletal muscle cells functions as the receptor for motor axon-derived Agrin, and upon Agrin binding Lrp4 activates the muscle-specific kinase (MuSK) receptor, initiating downstream signaling through Rapsyn eventually leading to the accumulation of acetylcholine receptor (AChR) clusters underneath the nerve terminal (Fig. 4k)^31,38–41^. Because of its role in synapse development as an obligate signaling receptor for Lrp4, we first tested whether MuSK is required for peripheral nerve regeneration. For this we examined peripheral nerve regeneration in *musk* null mutants^42^. Endpoint analysis of peripheral nerve regeneration at 48 hpt did not reveal any significant differences between *musk* mutants and wild-type siblings (Fig. 4a–d, i). During synapse formation, Agrin is the ligand that binds Lrp4 to activate MuSK^40,41^. Zebrafish *agrin* mutants have not previously been generated, so we used CRISPR/Cas9 genome editing^43^ to generate *agrin* mutants affecting the protein isoforms previously shown in mice to be critical for motoneuronal Agrin release and synapse formation (Supplementary Fig. 2a–b)^44^. As predicted, neuromuscular synapse development in zebrafish *agrin* mutants was severely disrupted compared to wild-type siblings (Supplementary Fig. 2c–f). In contrast, peripheral nerve regeneration in these *agrin* mutants was unaffected (Fig. 4e–h, j). Finally, Rapsyn is a key downstream effector of MuSK signaling^45,46^, and analysis of *rapsyn* mutants failed to reveal deficits in peripheral nerve regeneration (Supplementary Fig. 3). Thus, *lrp4* promotes regeneration through a non-canonical, Agrin/MuSK-independent signaling pathway.

### A truncated form of Lrp4 promotes nerve regeneration

**Fig. 5:**
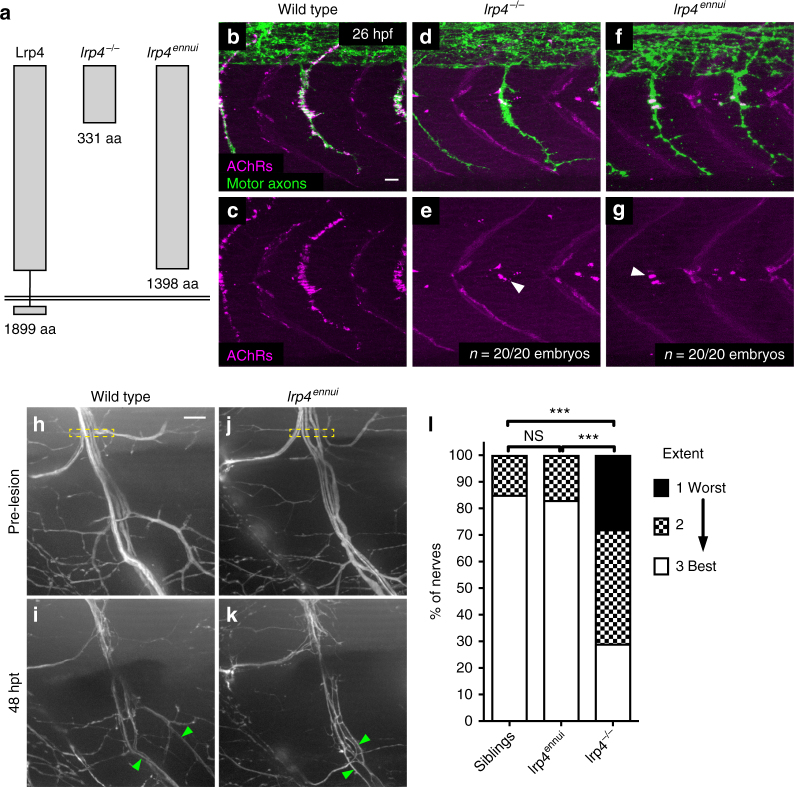
A truncated version of Lrp4 promotes nerve regeneration. **a** Schematic of *lrp4* alleles and their protein structures: wild-type full-length Lrp4 is shown at far left (1899 amino acids). *lrp4*^*−/−*^ allele encodes a protein of 331 amino acids. *lrp4*^*ennui*^ encodes a protein of 1398 amino acids that is predicted to contain almost the entire ectodomain but lack the transmembrane and intracellular domains. **b**–**g** Lateral views of wild type, *lrp4*^−/−^, and *lrp4*^*ennui*^ embryos at the stage when *en passant* neuromuscular synapses form (26 hpf) stained for acetylcholine receptors (AChRs; magenta) and motor axons (green). Scale bar is 10 µm. **b**–**c** In wild-type embryos, AChR clusters assemble beneath the entire motor axon. **d**–**g** In *lrp4* mutant and *lrp4*^*ennui*^ embryos there is a strong reduction in AChR clustering beneath the motor axon, with only a few AChR clusters observed (white arrowheads); *n* = 20 out of 20 embryos examined for each genotype. **h** Lateral view of pre-transection wild-type motor nerve labeled with GFP at 5 dpf. Transection site is marked by yellow box. Scale bar is 10 µm. **i** Forty-eight hours post transection in wild-type larvae, multiple fascicles have regenerated ventrally (marked by green arrowheads). **j** Lateral view of pre-transection motor nerve in *lrp4*^*ennui*^ labeled with GFP at 5 dpf. Transection site marked by yellow box. **k** Forty-eight hours post transection in *lrp4*^*ennui*^, multiple fascicles have regenerated ventrally (green arrowheads). **l** Quantification of nerve regeneration in wild-type larvae (*n* = 26 nerves from 13 larvae), *lrp4* mutants (*n* = 28 nerves from 13 larvae) and *lrp4*^*ennui*^ (*n* = 24 nerves from 9 larvae) at 48 h post transection. *lrp4*^*ennui*^ nerves regenerate to the same extent as wild-type sibling nerves (wild-type siblings “best” regeneration = 85% of nerves; *lrp4*^*ennui*^ “best” regeneration = 83% of nerves; *χ*^2^-test *p* = 0.1672, *χ*^2^ = 3.577, df = 2)

In addition to its role as a muscle membrane bound MuSK co-receptor, Lrp4 has also been shown to function in bone and tooth development, where it modulates Wnt signaling in part by releasing its extracellular domain, and hence sequestering Wnt antagonists^47–49^. Moreover, several reports have shown the Lrp4 ectodomain can be cleaved and that it retains biological activity^48,50–52^. We therefore wanted to explore the possibility that Lrp4 promotes peripheral nerve regeneration through an anchorage-independent mechanism. Previously, cloning of the zebrafish *ennui* mutant^53^ had revealed that the mutant phenotype is caused by a premature stop codon in Lrp4, just before the transmembrane domain (amino acid 1398 out of 1899), generating a truncated protein lacking membrane anchoring and the intracellular domain while leaving intact almost the entire extracellular domain (Fig. 5a). We first determined whether and to what degree this truncated Lrp4 protein had retained activity to induce neuromuscular synapses. For this we compared postsynaptic AChR cluster localization between wild type, *lrp4* null mutant and *lrp4*^*ennui*^ mutant embryos at 26 hpf when motor axons have formed *en passant* neuromuscular synapses with muscle cells (Fig. 5b**–**g)^53^. This revealed a very strong reduction of postsynaptic AChR clusters in *lrp4*^*ennui*^ mutants, identical to what we had previously reported for *lrp4* null mutant animals (Fig. 5d**–**g)^21^ and similar to what we observe in *agrin* mutants (Supplementary Fig. 2e, f). In contrast to the almost complete inability of *lrp4*^*ennui*^ to function in neuromuscular synapse development, peripheral nerve regeneration at 48 hpt was indistinguishable from that in wild-type animals, and in stark contrast to the phenotype observed in *lrp4* null mutants (Fig. 5h–l). Thus, a truncated form of Lrp4 lacking the transmembrane and cytoplasmic domains that lacks activity as a MuSK co-receptor is sufficient to promote regeneration. Combined with our findings that Lrp4 acts independently of MuSK signaling, this supports a model by which Lrp4 is repurposed independently from its role in synapse development to promote sustained axonal regeneration and regulate Schwann cell plasticity, possibly via interactions between these two cell types.

## Discussion

Lrp4 function has been studied most extensively in its role as the Agrin receptor expressed on skeletal muscle cells where Lrp4 signals through its obligate co-receptor MuSK^37,40^. Here we provide compelling in vivo evidence that Lrp4 promotes peripheral nerve regeneration independently of MuSK, Agrin, and its downstream effector Rapsyn (Fig. 4), all critical for neuromuscular synapse development in mammals and also in zebrafish^37,38,42,54^. Moreover, by using a mutant *lrp4* allele that lacks membrane anchoring and the intracellular domain but leaves the ectodomain mostly intact, we demonstrate that in zebrafish Agrin-dependent synapse formation requires membrane-bound Lrp4 to an even greater degree than in mouse mutants with an allele that encodes only the Lrp4 ectodomain^52^. The finding that Lrp4 membrane anchoring is dispensable for peripheral nerve regeneration strongly argues that Lrp4 promotes regeneration largely independent of its well-defined signaling pathway critical for neuromuscular synapse development.

How could Lrp4 promote peripheral nerve regeneration? Lrp4 is a member of the large family of low-density lipoprotein receptor-related proteins with distinct roles inside but also outside the nervous system^47,50,55–57^. Outside the nervous system, Lrp4 plays critical roles in bone, tooth, and limb development, primarily by modulating the Wnt and BMP signaling pathways^47,49,56^, and both the BMP antagonist Wise and the Wnt antagonist Dickkopf-1 bind Lrp4^47,58^. While Wnt signaling has been shown to play a functional role in CNS regeneration^59,60^ its role in peripheral nerve regeneration is not well understood, although a recent report suggests that Wnt/β-Catenin signaling regulates Schwann cell proliferation and migration after sciatic nerve injury^61^. Similarly, BMP signaling can promote axonal regeneration, although most studies have focused on spinal cord injury rather than peripheral nerve injury^62,63^. Existing zebrafish mutants for both signaling pathways will be invaluable in establishing potential roles for BMP and/or Wnt signaling in Lrp4-dependent peripheral nerve regeneration.

During development, pioneer neurons form the initial axonal scaffold used by later outgrowing axons. Pioneering neurons have been identified in many organisms ranging from sensory neurons in grasshopper embryos to subplate neurons in the mouse cerebral cortex^22,64^. Sequential axonal outgrowth is thought to simplify the many challenges of axonal pathfinding by enabling the majority of axons to simply extend along a pre-existing axonal path. While in some cases pioneer axons appear largely dispensable for follower axons to find their targets^65^, in many cases pioneering axons are necessary for follower axons to innervate their appropriate targets^25,66,67^.

Yet despite their importance in development, whether pioneer axons play a role in regeneration or even whether regenerating axons are organized into pools of pioneer and follower axons is not well understood. We find that after complete nerve transection and a period of intense neurite extension and retraction (Supplementary Movie 2, open blue arrowheads), frequently a single axon emerges from the proximal nerve stump to pioneer a regenerative path across the injury site and back to the ventral myotome. Multiple later emerging axons join the pioneering axon and extend with about twice the speed of the pioneering axon across the injury gap, consistent with the idea that pioneering axons provide a growth-promoting, adhesive substrate. In *lrp4* mutants pioneer axons successfully navigate across the injury gap and toward their original targets while follower axons appear unable to join the pioneering axon and frequently fail to cross the injury gap, eventually leading to reduced nerve regeneration (Figs. 1 and 2). Based on these results it is tempting to speculate that during regeneration pioneer axons are important to promote the growth of later emerging follower axons, and that *lrp4* produced by either pioneer or follower axons is critical for this interaction. However, our findings that *lrp4* expression in motor axons is not sufficient to restore axonal regeneration (Fig. 2) strongly suggest an axon extrinsic mechanism.

Independent of the precise underlying mechanisms, our results demonstrate that like in development the majority of regenerating axons take advantage of and extend along a pre-existing path, consistent with the idea that developmental strategies are reutilized during regeneration. Importantly, in development axonal outgrowth of pioneering primary motor axons and follower secondary motor axons is unaffected in *lrp4* mutants^21^. Thus, our analysis reveals that regenerating axons are organized into temporally segregated pools of pioneer and follower axons, and uncovers *lrp4*-dependent functional heterogeneity between pioneering and follower axons dedicated to regeneration.

A hallmark of peripheral nerve regeneration is the remarkable plasticity of Schwann cells. Already described by Santiago Ramon y Cajal^1^, upon nerve injury Schwann cells undergo a reversible transformation characterized by extensive morphological changes. The initial transformation into a denervated, activated Schwann cell occurs in response to nerve injury, and reflects the conversion into a more immature cell state. Activated Schwann cells are characterized by the loss of myelin sheaths, reactivation of developmental genes and their ability to divide and migrate^3,16^. While this first transformation has been studied extensively, the molecular mechanisms that trigger the morphological transformation of denervated, activated Schwann cells back to their pre-injury appearance is not well understood. We show that in *lrp4* mutants Schwann cell development and injury-induced transformation into an activated state are unaffected, strongly arguing that *lrp4* is dispensable for the molecular mechanisms and cellular forces underlying general Schwann cell plasticity. Unlike in wild-type animals, Schwann cells in *lrp4* mutants fail to return to their original morphology despite the presence of pioneering axons making apparent contacts with denervated Schwann cells (Fig. 3k). This strongly argues against the notion that contacts between regenerating axons and denervated Schwann cells are sufficient to trigger changes in Schwann cell morphology characteristic of their re-differentiation.

The analysis of *lrp4* mutants reveals a clear defect in the ability of denervated Schwann cells to resume their more differentiated morphology, consistent with several interpretations. One interpretation is that the Schwann cell phenotype is simply a secondary consequence of the reduced number of regenerating axons. While we cannot exclude this possibility, we favor an alternative possibility that *lrp4* functions in glial cells, specifically in denervated Schwann cells to coordinate changes in Schwann cell morphology with axonal regeneration. There is precedent for *lrp4* functioning in astrocytes where *lrp4* regulates synaptic transmission^57^. Moreover, during development *lrp4* functions not only in muscle cells as a receptor for postsynaptic differentiation but the *lrp4* ectodomain also acts as a muscle-derived retrograde signal that binds preferentially to the distal portion of motor axons^50^. Such bidirectional signaling might also occur during axonal regeneration: as pioneering axons cross the injury gap and contact denervated Schwann cells this might activate Lrp4 signaling within Schwann cells, triggering the morphological changes associated with re-differentiation to a more mature ensheathing Schwann cell.

Concomitantly, contacts between the pioneering axon and denervated Schwann cells might trigger cleavage of the Lrp4 ectodomain from the Schwann cell surface and like in development the Lrp4 ectodomain might bind to axons to act as a retrograde signal. Binding of the Lrp4 ectodomain to pioneering motor axons could trigger a mechanism to make them a growth-promoting, adhesive substrate for follower axons. Such a scenario would explain why in *lrp4* null mutants pioneering axons regrow while follower axons fail to extend along the pioneer axon and instead frequently stall. It would also be consistent with our findings that *lrp4* expression in motor axons is not sufficient to restore axonal regeneration (Fig. 2) and that in *lrp4*^*ennui*^ mutants encoding only the ectodomain, regeneration is unaffected (Fig. 5). Independently of the underlying mechanism, our results uncover a novel role for *lrp4* in promoting peripheral nerve regeneration. We demonstrate that Lrp4 function has been co-opted in regeneration independently of the well-documented MuSK signaling cascade in neuromuscular synapse formation. Finally, we uncover the existence of pioneer and follower axons in peripheral nerve regeneration, and demonstrate differential sensitivity to *lrp4* function, revealing the existence of molecular diversity between regenerating axons.

## Methods

### Zebrafish strains and animal care

Transgenic lines were generated in the Tübingen or TLF genetic background, and all fish used were maintained as previously described^68^. The following transgenic lines were used: *Tg(mnx1:GFP)*^*ml2*^, *Tg(sox10:mRFP)*^*vu234*^, *Tg(mnx1:Lrp4-GFP)*^*p169Tg*^, *Tg(α-actin:Lrp4-GFP)*^*p159Tg*^. The following mutant strains were used: *lrp4*^*p184*^ ^21^, *lrp4*^*mi36*^, textually referred to as *lrp4*^*ennui*^^53^, *musk*^*tbb72*^^42,69^, *two*^*th26*^, textually referred to as *rapsyn*^46,69^, and *agrin*^*p168*^. We conducted all experiments using zebrafish according to animal protocols that were approved by the University of Pennsylvania IACUC regulatory standards.

### Generation of agrin^p168^ mutants

The zebrafish *agrin* (XM_009296860.2) exon 31 splice donor site was targeted for CRISPR-mediated mutagenesis in order to generate exclusively Z- *agrin* transcripts in vivo, which has been shown to eliminate the AChR clustering ability of Agrin^44^. To generate templates for sgRNA transcription, 16-bp complementary *agrin*-specific oligonucleotides were ordered from IDTDNA, with BsaI overhangs added to allow the oligos to be annealed and then ligated into pDR274 (Forward *agrin* oligo: 5′-TAGGCAGAGGAGAGGCGCCGGT-3′; Reverse *agrin* oligo: 5′-AAACACCGGCGCCTCTCCTCTG-3′). The sgRNAs in pDR274 were transcribed using the T7 MegaShortScript kit (Ambion). Fifty picograms sgRNA and 80 pg Cas9 protein (PNA Bio) were co-microinjected into one-cell stage TLF embryos. Double-stranded DNA breaks in *agrin* exon 31 of G0 injected embryos were confirmed by PCR and high-resolution melt analysis, and G0 embryos were raised. Heterozygous F1 carrier alleles were identified and characterized by PCR and sequencing, and transcripts were characterized by sequencing from pools of mutant and sibling cDNA.

### Molecular identification of ennui gene

By using recombination mapping and PCR-scorable, polymorphic CA repeats the *ennui* mutation was mapped to a 1 cM region on chromosome 7. Examination of over 2500 meiotic events identified a single-non-recombinant CA repeat marker in an intron of the *lrp4* gene. Subsequent RT-PCR analysis using mRNA from both wild type and *ennui* embryos, revealed a single-point mutation in the *lrp4* open reading frame that converted Tyrosine 1398 to a stop codon. This mutation was confirmed by sequencing PCR-amplified genomic DNA in the region of the mutation from wild type and mutant embryos.

### Spinal motor nerve transection assays

Complete MicroPoint (Andor Technology) laser-mediated transection and live-cell imaging of peripheral motor nerves was performed as previously described^27–29^. Nerve regeneration scoring at 48 hpt was performed as follows: image brightness and contrast were altered in the same way for every image across all experiments, and 48 hpt images were anonymized for blind scoring. Using our previously established regeneration scoring rubric^28^, we assigned a regeneration score to each transected nerve based on the number of regenerated fascicles that regrew to the ventral end of the hemisegment. “Best” regeneration indicates two or more distinct regenerated fascicles; “moderate” indicates one regenerated fascicle; “worst” indicates zero regenerated fascicles that regrew to the ventral end of the hemisegment. Images were subsequently un-blinded and regeneration scores were recorded in GraphPad Prism for statistical analysis. Fisher’s exact tests or *χ*^2^-tests were used to compare the extent of regeneration between different genotypes.

### Sparse neuronal labeling

A DNA vector encoding *mnx1:mKate* was injected into one-cell-stage *Tg(mnx1:GFP)*^*ml2*^ embryos as previously described^70^. Embryos were sorted for mKate signal in individual motor neurons at 24 hpf, and GFP-expressing motor nerves containing a small subset of mKate-expressing axons were transected at 5 dpf and imaged as described above.

### Whole-mount immunohistochemistry and imaging

To label acetylcholine receptor clusters and motor axons, zebrafish embryos at 26–28 hpf were permeabilized in 1 mg/ml collagenase in phosphate-buffered saline for 7 min, rinsed in 1× PBS, and stained with fluorescently conjugated ɑ-bungarotoxin (1:1000; Molecular Probes, Eugene, OR) and the znp-1 antibody (1:200; Developmental Studies Hybridoma Bank). Embryos were mounted in Vectashield (Vector Laboratories) and imaged in 1 µm sections using a ×40 or ×60 objective on a Zeiss LSM710 confocal microscope. Image stacks were adjusted for brightness and contrast in Fiji. An anti-Sox10 antibody (1:2000; gift from S. Kucenas) was used to label Schwann cell nuclei in 5 dpf and 6 dpf larvae as previously described^28^. Larvae were mounted in Vectashield, and imaged in 1 µm sections using a ×60 objective on a Zeiss LSM710 confocal microscope. Image stacks were adjusted for brightness and contrast in Fiji, and Schwann cell nuclei were manually counted in each *z*-plane of an image. The results were recorded in GraphPad Prism for statistical analysis: two-tailed *t*-tests were performed to compare Schwann cell numbers pre- and post transection.

### Data availability

The datasets generated and analyzed during the course of this study are available from the corresponding author upon reasonable request.

## Electronic supplementary material

Supplementary Information

Description of Additional Supplementary Files

Supplementary Movie 1

Supplementary Movie 2

Supplementary Movie 3

Supplementary Movie 4

Supplementary Movie 5
