# Supplementary material for: The synaptic receptor Lrp4 promotes peripheral nerve regeneration
Source: Nat Commun. 2018 Jun 19;9:2389. doi: 10.1038/s41467-018-04806-4 (PMC6008306; doi:10.1038/s41467-018-04806-4)
Supplement: Supplementary file 1 — Supplementary Information [file 41467_2018_4806_MOESM1_ESM.pdf]

# The synaptic receptor Lrp4 promotes peripheral nerve regeneration

Gribble et al.

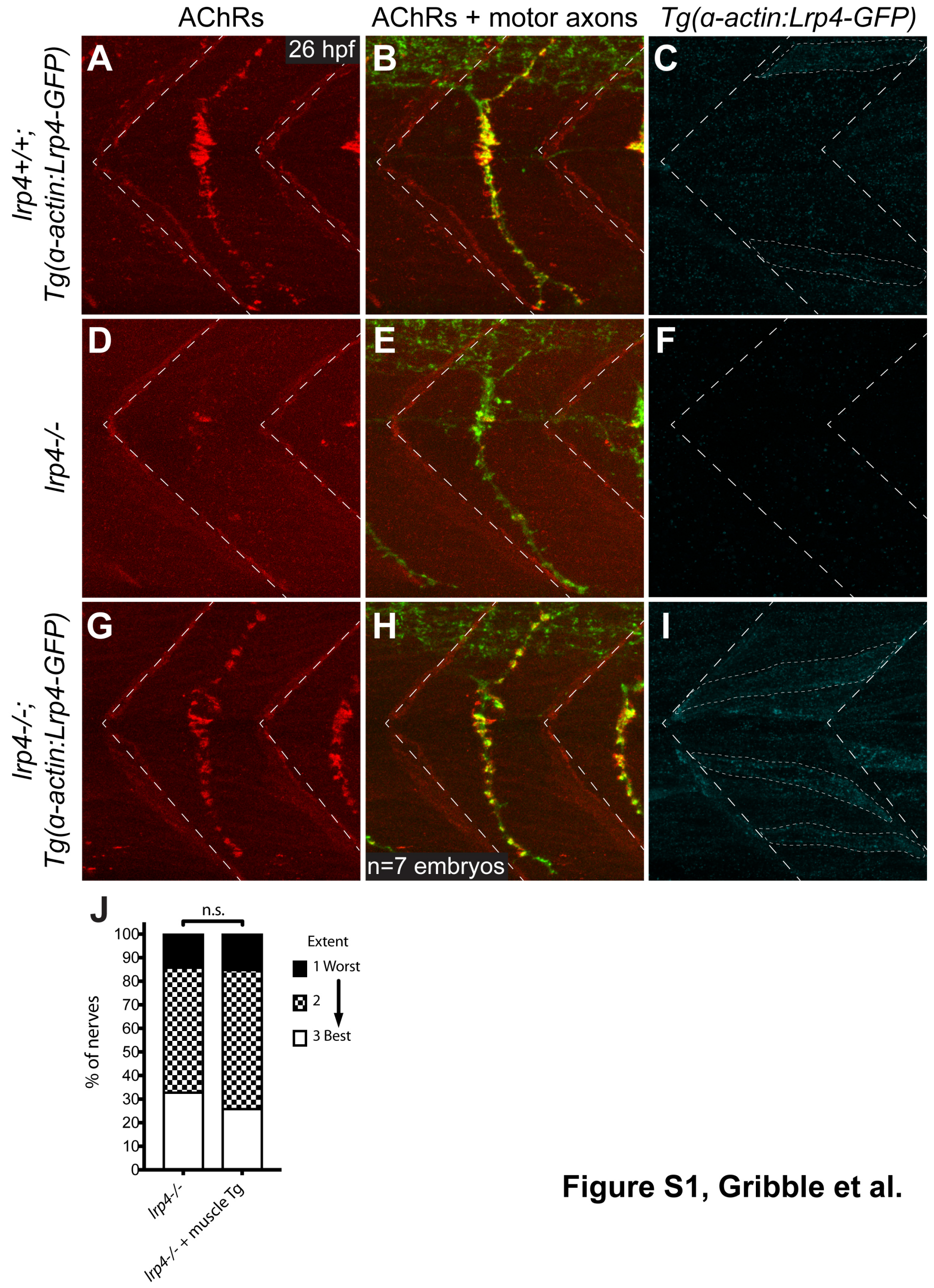


**Figure S1, Gribble et al.**

**Supplementary Figure 1. Selective expression of *lrp4* in muscle cells rescues neuromuscular synaptic defects in *lrp4* mutants**

(A) Lateral view of single hemisegment in a 26 hpf wild-type zebrafish embryo expressing *Tg(α-actin:Lrp4-GFP)*, showing normal distribution of acetylcholine receptors (AChRs) in the center of the hemisegment. (B) Merged image of AChRs and motor axons labeled with znp-1 antibody, showing AChRs clustered beneath motor axon terminals. (C)

Localization and expression of *Tg(α-actin:Lrp4-GFP)* in wild-type muscle cells. Lrp4-GFP localizes in puncta distributed throughout each muscle cell. (D,E) *lrp4* mutants at 26 hpf show a dramatic reduction in AChR clusters and neuromuscular synapses, but motor axons extend normally. (F) No Lrp4-GFP signal is observed in this mutant, and the muscle rescue transgene is not detectable by PCR. (G,H) *lrp4* mutants expressing *Tg(α-actin:Lrp4-GFP)* show AChR clusters and neuromuscular synapses at 26 hpf that appear wild-type (n=7 embryos). (I) Expression of *Tg(α-actin:Lrp4-GFP)* in muscle cells of *lrp4* mutant embryo. (J) Quantification of nerve regeneration in *lrp4* mutant larvae and *lrp4* mutant larvae expressing the muscle-specific *α-actin:Lrp4-GFP* transgene. At 48 hpt, *lrp4* mutant larvae expressing *Tg(α-actin:Lrp4-GFP)* regenerate poorly (n=27 nerves), like *lrp4* mutant larvae (n=21 nerves; Chi-square test p=0.8505; Chi-square=0.3238, df=2).

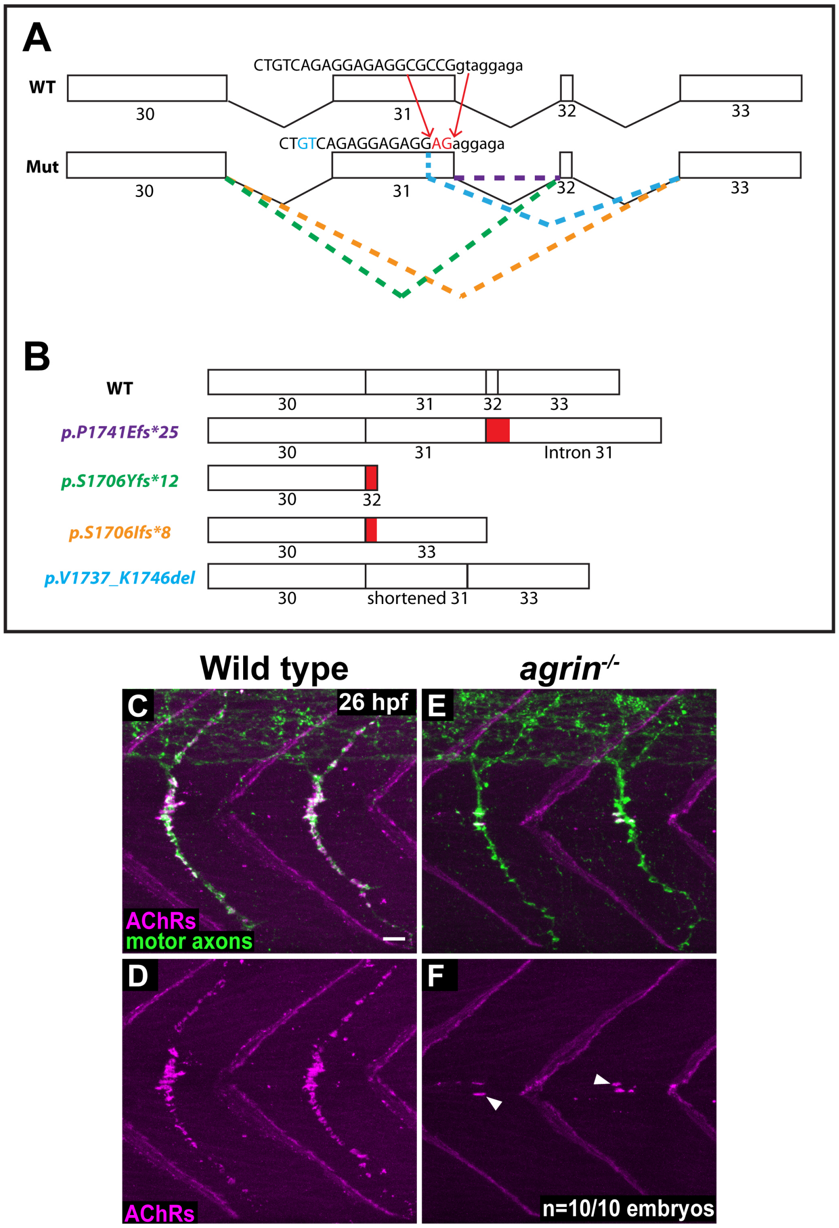


Figure S2, Gribble et al.

**Supplementary Figure 2. Zebrafish *agrin* is required for neuromuscular synapse formation**

(A) Summary of transcripts generated through CRISPR/Cas9 injection. The allele used for all experiments was a 7bpDEL;2bpINS at the end of exon 31, which resulted in loss of the exon 31 splice donor site. Wild type sequence is flanked by red arrows, and the genomic loss is marked in the mutant sequence just below. Sequencing cDNA from pools of mutants and siblings yielded four predominant transcripts, whose alternative splicing is shown using green, orange, blue, and purple dashed lines. (B) Exon structure and

resulting protein sequence for wild type *agrin* and the four predominant transcripts identified from sequencing cDNA. Three of four transcripts result in a frameshift and premature stop codon (purple, green, and orange transcripts), while one transcript is an in-frame deletion (blue transcript). All four transcripts result in severe swimming defects in zebrafish larvae at 36 hpf (data not shown). (C-D) At 26 hpf in wild type embryos, neuromuscular synapses (AChR labeling in magenta) are abundant beneath motor axons (green). (E-F) In *agrin* mutants, neuromuscular synapses are significantly reduced; white arrowheads show residual AChR clusters on the muscle cells at the horizontal myoseptum. N = 10 out of 10 embryos analyzed.

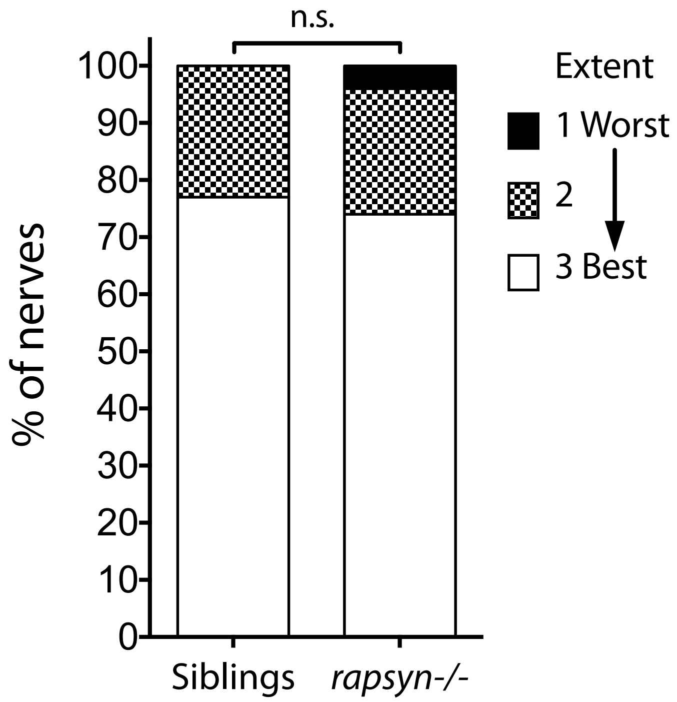


**Supplementary Figure 3. Zebrafish *rapsyn* is dispensable for motor axon regeneration.**

Quantification of nerve regeneration in wild type siblings (n=22 nerves from 14 larvae) and *rapsyn* mutants (n=27 nerves from 18 larvae) at 48 hours post-transection. *rapsyn* mutant motor nerves regenerate as well as wild type sibling motor nerves (Chi-square p=0.6595, Chi-square=0.8326, df=2).
