# Supplementary material for: The synaptic receptor Lrp4 promotes peripheral nerve regeneration
Source: Nat Commun. 2018 Jun 19;9:2389. doi: 10.1038/s41467-018-04806-4 (PMC6008306; doi:10.1038/s41467-018-04806-4)
Supplement: Supplementary file 2 — Description of Additional Supplementary Files [file 41467_2018_4806_MOESM2_ESM.pdf]

Description of Additional Supplementary Files

File Name: **Supplementary Movie 1**
Description: **Multiple follower axons fasciculate with the pioneer axon.** Lateral view of motor axon regeneration starting about 10 hours post-transection in a wild type sibling larva expressing GFP in all motor neurons. Proximal stump of the nerve is shown at the top of the image, from which axon sprouts extend and retract. At ~11 hpt, a pioneer axon extends from the proximal stump, crosses the injury gap, and grows ventrally along the original nerve trajectory (magenta arrow). Subsequently three follower axons recognize the pioneer and fasciculate with it to grow ventrally (green arrows).

File Name: **Supplementary Movie 2**
Description: **A single axon crosses the injury gap and pioneers the regenerative path for subsequent follower axons.** Lateral view of motor axon regeneration starting 7 hours post-transection (hpt) in a wild type sibling larva expressing GFP in all motor neurons and mKate in single motor axon. The proximal nerve stump is visible at the top of the movie, and from the start of imaging GFP- and mKate-positive axon sprouts extend and retract in the area around the injury site (open blue arrowheads). At ~11 hpt, a pioneering axon labeled with mKate extends from the proximal stump, crosses the injury gap, and grows ventrally along the original nerve trajectory (denoted with magenta arrowheads). At ~18 hpt, a GFP-positive follower axon (green arrowheads) crosses the injury gap and fasciculates with the pioneer axon. Images were captured every 10 minutes for 13 hours (from 7 hpt to 20 hpt).

File Name: **Supplementary Movie 3**
Description: ***lrp4* is dispensable for pioneer axon regrowth but required for follower axon regrowth.** Lateral view of motor axon regeneration starting about 10 hours post-transection in *lrp4* mutant larva expressing GFP in all motor neurons. A pioneering axon (magenta arrow) has almost fully extended when imaging begins. Proximal stump of the nerve is shown at the top of the image, and axon sprouts (blue arrows) extend and retract in the area around the injury site throughout the course of imaging. By the end of imaging period at 20 hours post-transection, no follower axons have fasciculated with the pioneer axon and regenerated ventrally.

File Name: **Supplementary Movie 4**
Description: **Time lapse imaging of Schwann cell morphology changes during early regeneration in a wild type sibling larva.** Lateral view of motor nerve regeneration starting 9 hours post-transection (hpt) in a wild type sibling expressing GFP in all motor neurons and mRFP in all Schwann cell membranes. When the movie starts at 9 hpt, Schwann cell membranes are discontinuous and granular as they de-differentiate and engulf axonal debris (yellow arrows from 540-590 minutes). As axons start regrowing (green arrowhead) around 11 hpt, Schwann cell membranes begin to flatten and re-extend and revert to their pre-injury morphology (yellow arrows).

File Name: **Supplementary Movie 5**
Description: **Time lapse imaging of Schwann cell morphology during early regeneration in *lrp4* mutant larva.** Lateral view of motor nerve regeneration 7 hours post-transection (hpt) in *lrp4* mutant expressing GFP in all motor neurons and mRFP in all Schwann cells to label Schwann cell membranes. When the movie starts at 7 hpt, Schwann cell membranes are discontinuous and granular as they de-differentiate and engulf axonal debris (yellow arrows). By the end of imaging at ~19 hpt, Schwann cell membranes have remained granular and discontinuous, and do not revert to their pre-injury morphology (yellow arrows).
